# The Surface Coating of Commercial LiFePO_4_ by Utilizing ZIF-8 for High Electrochemical Performance Lithium Ion Battery

**DOI:** 10.1007/s40820-017-0154-4

**Published:** 2017-09-25

**Authors:** XiaoLong Xu, CongYu Qi, ZhenDong Hao, Hao Wang, JinTing Jiu, JingBing Liu, Hui Yan, Katsuaki Suganuma

**Affiliations:** 1 The College of Materials Science and Engineering, Beijing University of Technology, Beijing, 100124 People’s Republic of China; 2 The Institute of Scientific and Industrial Research, Osaka University, Osaka, Japan

**Keywords:** LiFePO_4_, Zeolitic imidazolate frameworks-8, Surface coating, Cathode, Lithium ion battery

## Abstract

The requirement of energy-storage equipment needs to develop the lithium ion battery (LIB) with high electrochemical performance. The surface modification of commercial LiFePO_4_ (LFP) by utilizing zeolitic imidazolate frameworks-8 (ZIF-8) offers new possibilities for commercial LFP with high electrochemical performances. In this work, the carbonized ZIF-8 (C_ZIF-8_) was coated on the surface of LFP particles by the in situ growth and carbonization of ZIF-8. Transmission electron microscopy indicates that there is an approximate 10 nm coating layer with metal zinc and graphite-like carbon on the surface of LFP/C_ZIF-8_ sample. The N_2_ adsorption and desorption isotherm suggests that the coating layer has uniform and simple connecting mesopores. As cathode material, LFP/C_ZIF-8_ cathode-active material delivers a discharge specific capacity of 159.3 mAh g^−1^ at 0.1C and a discharge specific energy of 141.7 mWh g^−1^ after 200 cycles at 5.0C (the retention rate is approximate 99%). These results are attributed to the synergy improvement of the conductivity, the lithium ion diffusion coefficient, and the degree of freedom for volume change of LFP/C_ZIF-8_ cathode. This work will contribute to the improvement of the cathode materials of commercial LIB.

**Electronic supplementary material:**

The online version of this article (doi:10.1007/s40820-017-0154-4) contains supplementary material, which is available to authorized users.

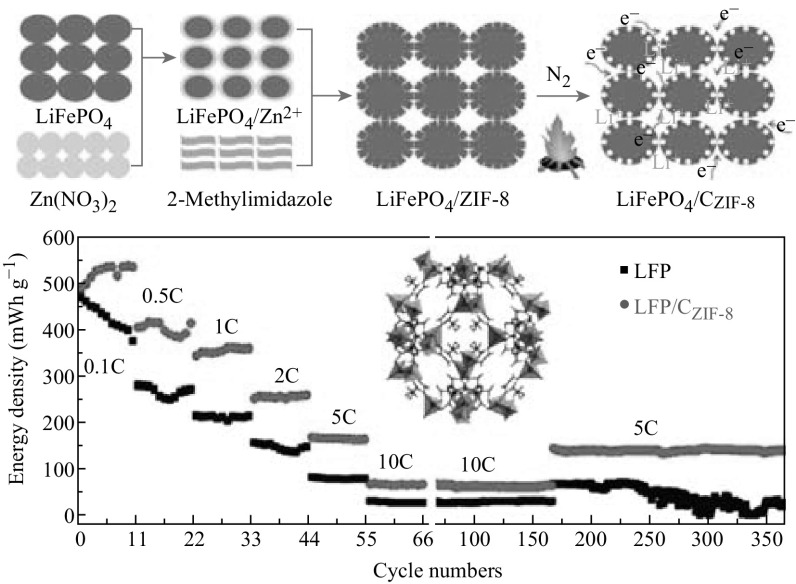

## Highlights

- A surface modification layer, which has 10 nm with metal zinc and graphite-like carbon, was synthesized on commercial LiFePO_4_ (LFP) using ZIF-8.
- As-prepared LFP/C_ZIF-8_ possesses prominent electrochemical performances with a discharge specific capacity of 159.3 mAh g^−1^ at 0.1C and a discharge specific energy of 141.7 mWh g^−1^ after 200 cycles at 5.0C.

## Introduction

As the world’s population grows and the natural non-renewable resource consumes, developing the lithium ion batteries (LIBs) with high electrochemical performances become increasingly important [1–3]. Recently, LIBs with LiFePO_4_ (LFP) as cathodes have matured significantly and serviced the markets of modern electronics and electric vehicles [4–6]. However, the LFP with high specific energy and high cycle stability is still facing a great challenge, because the electron/ion transfer and structural stability of commercial electrode materials cannot meet the harsh ultrahigh-rate working environment [7, 8]. Therefore, it is the assiduous goal to develop advanced electrode materials with high performances.

High specific energy electrodes are highly related to voltage platforms [9, 10] and specific capacities of electrodes [11, 12]. For LFP cathode material, we have demonstrated that the increased voltage platform depends on the improvement of electrode conductivity [13]. In the past study, it was demonstrated that the carbon coating was an effective strategy to improve the conductivity of electrode material [14]. In general, the coating layer is amorphous carbon [15, 16], because the crystallization temperature of carbon is much higher than the crystal growth temperature of LFP. Meanwhile, the *sp* ^2^ carbon material is conducive to the transmission of electrons [17], which may be beneficial to improve the conductive electrode material. Recently, some progress has been made in the surface coating of electrode material by using crystalline carbonaceous material at low temperature [1]. Moreover, metal elements are also used to improve the electrode conductivity due to their free electrons [18]. The improvement of the conductivity also has a significant effect on the specific capacity of the electrode material [1, 18]. These studies provide a theoretical basis for the improvement of specific energy of LFP cathode material.

The high cycle stability mainly depends on the structural stability of the electrode material at different charge and discharge current rates [19, 20]. For LFP cathode material, both volume shrinkage (charge/lithium ion deintercalation) and expansion (discharge/lithium ion intercalation) will lead to structural damage and thus cause capacity decline [21]. It is demonstrated that the suitable coating layer with porous structure can effectively improve the structural stability of the active material by buffering volume change [22, 23]. This may be an appropriate modification method for commercial electrode materials.

The surface coating plays an important role in the improvements of the electrochemical performances. The choice of coating materials is the key to improve the electrode performance. Zeolitic imidazolate frameworks-8 (ZIF-8) is a class of porous materials, which attracted considerable interest due to its regular polyhedral morphology and ordered pore structure [24, 25]. The pyrolysis products of ZIF-8 under anaerobic conditions are porous carbon materials with high specific surface areas and high conductivities [26, 27]. Torad et al. [28] and Zhang et al. [29] reported that graphite-type carbon was prepared at 800 °C by using ZIF-8 as raw materials, and they also proved that crystalline carbon can be prepared at lower temperature. Moreover, the formation of metallic zinc annealed in an inert atmosphere is also conducive to the improvement of conductivity. Although ZIF-8 as coating material used to modify LIB anodes and showed excellent electrochemical performances [30, 31], ZIF-8 has not been used for surface coating of LFP cathode so far. Therefore, it is necessary to explore the effect of ZIF-8 in LFP cathode coating for improving the electrochemical performances of commercial LIB.

In this work, we modify the commercial LFP by the growth and carbonization of ZIF-8 on the surface of LFP particles. The prepared LiFePO_4_/carbonized ZIF-8 (LFP/C_ZIF-8_) was demonstrated evidently to improve the electrochemical performances compared with pristine commercial LFP materials.

## Experimental

### Coating Process

The raw materials used in this experiment include methanol (99.5%, Beijing Chemical Works), zinc nitrate (99%, Tianjin Fuchen Chemical Reagent Factory), 2-methylimidazole (99%, Sinopharm Group Chemical Reagent Co., Ltd.), and commercial LiFePO_4_ powder (Qinghai Taifeng First Lithium Technology Co., Ltd.).

The coating processes were performed as described below: LFP was dispersed into methanol to form a uniform slurry by ultrasonic. Then, zinc nitrate and 2-methylimidazole were dissolved into the above slurry by magnetic stirring for 1 h. The mixture was aged at room temperature for 24 h and the gray powders were precipitated. The ratio of LFP dispersion: zinc nitrate: 2-methylimidazole was 100 mL:1.029 g:1.314 g. And the ratio of LFP: methanol was 8(g):x(mL) (*x* = 0, 188, 282, 376). The powders were washed very carefully with methanol and annealed at 500/600/700/800/900 °C with a heating rate of 5 °C min^−1^ under nitrogen flow to obtain the LFP/C_ZIF-8_ samples. As the blank sample, the ZIF-8 was synthesized using the same method without LFP.

### Characterization

The phase compositions of the synthesized products were analyzed by X-ray diffraction (XRD) employing a Cu-K*α* X-ray diffractometer (D8 ADVANCE Bruker AXS). The morphologies of grains were characterized by transmission electron microscopy (TEM) with an energy dispersive spectrometer (EDS), TEM images were obtained using a Philips Tecnai 20U-TWIN microscope. Surface composition of the sample is investigated using X-ray photoelectron spectroscopy (XPS, ESCALAB 250 XPS using Al K*α* (1486.6 eV) radiation). The N_2_ adsorption and desorption isotherms and Barrett–Joyner–Halenda pore-size-distribution were obtained at 77 K using an automatic surface area analyzer (Micromeritics, Gemini V2380, USA) under continuous adsorption conditions. Raman spectroscopic analysis was performed with a 532 nm FILTER In-Via Raman microscopic instrument.

### Electrochemical Evaluation

The charge and discharge performances were determined with CR 2032 coin cells. The cathode materials were prepared by mixing the LFP/C_ZIF-8_ with acetylene black and poly-(vinylidene fluoride) in a weight ratio of 8:1:1 in *N*-methyl pyrrolidone to ensure homogeneity. Then, the slurry was coated on an Al-foil with about 0.02 mm thickness, dried under the air atmosphere at 60 °C for 5 h and the vacuum atmosphere at 120 °C for 10 h, and cut into circular strips of 15 mm in diameter. The loading densities are ∼2.3 mg cm^−2^. The cells were assembled in a glove box filled with high purity argon, where lithium metal was used as an anode, polypropylene film as a separator, and 1 M LiPF_6_ as an electrolyte consisting of ethylene carbonate/dimethyl carbonate/ethylene methyl carbonate in a volume ratio of 1:1:1 (1 M LiPF_6_/EC/DEC/EMC) as lithium ion electrolyte. Under each condition, five identical samples were synthesized.

The charge and discharge performances of the LFP/C_ZIF-8_ were tested on a channels battery analyzer (CT3008W) at different current densities between 2.5 and 4.2 V cut-off voltage using the coin cells. The electrochemical impedance spectroscopy (EIS) measurements were performed on a PARSTAT 4000 electrochemical workstation. EIS was also recorded with frequencies ranging from 100 kHz to 10 mHz and an AC signal of 5 mV in amplitude as the perturbation. All the tests were performed at room temperature.

## Results and Discussion

**Fig. 1:**
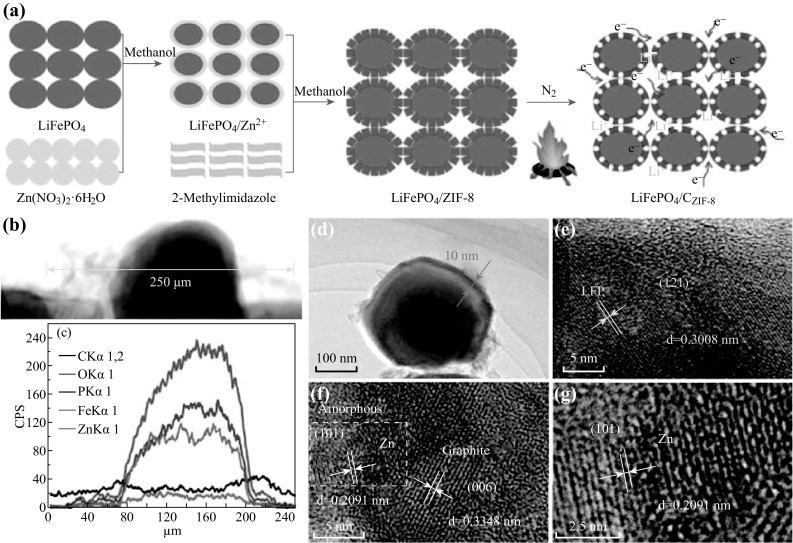
**a** Schematic illustration of the synthesis process. **b** TEM image, **c** line scanning EDS, **d** surface coating layer structure of LFP/C_ZIF-8_. Regular lattice fringes of **e** LFP, **f** graphite and **g** Zn crystal

Figure 1a shows a schematic illustration of the synthesis route for the LFP/C_ZIF-8_. The SEM image shows the average size of LFP particles is about 4 μm in Fig. S1. In the methanol solution, the uniformly dispersed micron-scale LFP particles provide more positions for non-uniform nucleation of ZIF-8 [32]. When zinc nitrate and 2-methylimidazole are dissolved in methanol, ZIF-8 nuclei are rapidly formed on the surface of LFP particles. After aging, the ZIF-8 particles grow up and the annealing precursors (LFP/ZIF-8) are formed. After high-temperature annealing under nitrogen atmosphere, the organic ligands are pyrolyzed and crystallized to form graphite-like carbon. At the same time, a reducing atmosphere is formed, and the zinc ions are reduced to elemental zinc. The pore formed by the pyrolysis carbonization of the imidazole ligand is preserved due to the slow heating rate. The XRD (Fig. S2) patterns of samples show that the peaks of all samples are indexed to the LiFePO_4_ phase, and it indicates that the surface coating does not alter the crystal structure of LFP. Moreover, as shown in Fig. S3a, all peaks are consistent with the ZIF-8 nanocrystals, which prove that ZIF-8 can be synthesized in the methanol solution. The electrochemical performances of LFP/C_ZIF-8_ cathode materials are evaluated and compared by charge and discharge tests using CR2032 coin cells (Fig. S4). The results illustrate that the optimal ratio of LFP/methanol is 8 g:282 mL) and the optimal heat treatment temperature is 800 °C).

The TEM and line scanning EDS analysis were employed to probe the composition and structure of the coating layer. The yellow line in Fig. 1b marks the line scanning EDS test range from 0 to 250 μm. The element distribution curve in Fig. 1c shows that there are C and Zn in the coating layer on the particle surface because of the existent curves of carbon and Zn in the range of 60–200 μm. The Zn content is less because the annealing temperature is close to its boiling point (908 °C), it causes the volatilization of metal Zn at 800 °C. A nano-scaled LFP/C_ZIF-8_ particle with the particle size of about 300 nm is shown in Fig. 1d. There is a 10 nm coating layer on the surface of LFP/C_ZIF-8_ particle. Figure 1e shows the clear regular lattice fringes with a *d*-spacing of 0.3008 nm which corresponds to the (121) plane of LFP. The regular lattice fringes with the *d*-spacing of 0.3348 nm correspond to the (006) plane of graphite, and the lattice fringes of Zn were also found in the white dashed box (Fig. 1f). The enlarged image displays that the *d*-spacing of 0.2091 nm corresponds to the (101) plane of elemental zinc. These results demonstrate the in situ growth of elemental zinc and graphite-like carbon on LFP surface. Moreover, the XPS results also confirm the existing of elemental zinc (Fig. S5). Two peaks at 1021.78 eV (2*p*3/2) and 1044.88 eV (2*p*1/2) and the peak splitting of 23.1 eV assure the presence of elemental zinc, which is beneficial to enhance the conductivity of LFP/C_ZIF-8_.

**Fig. 2:**
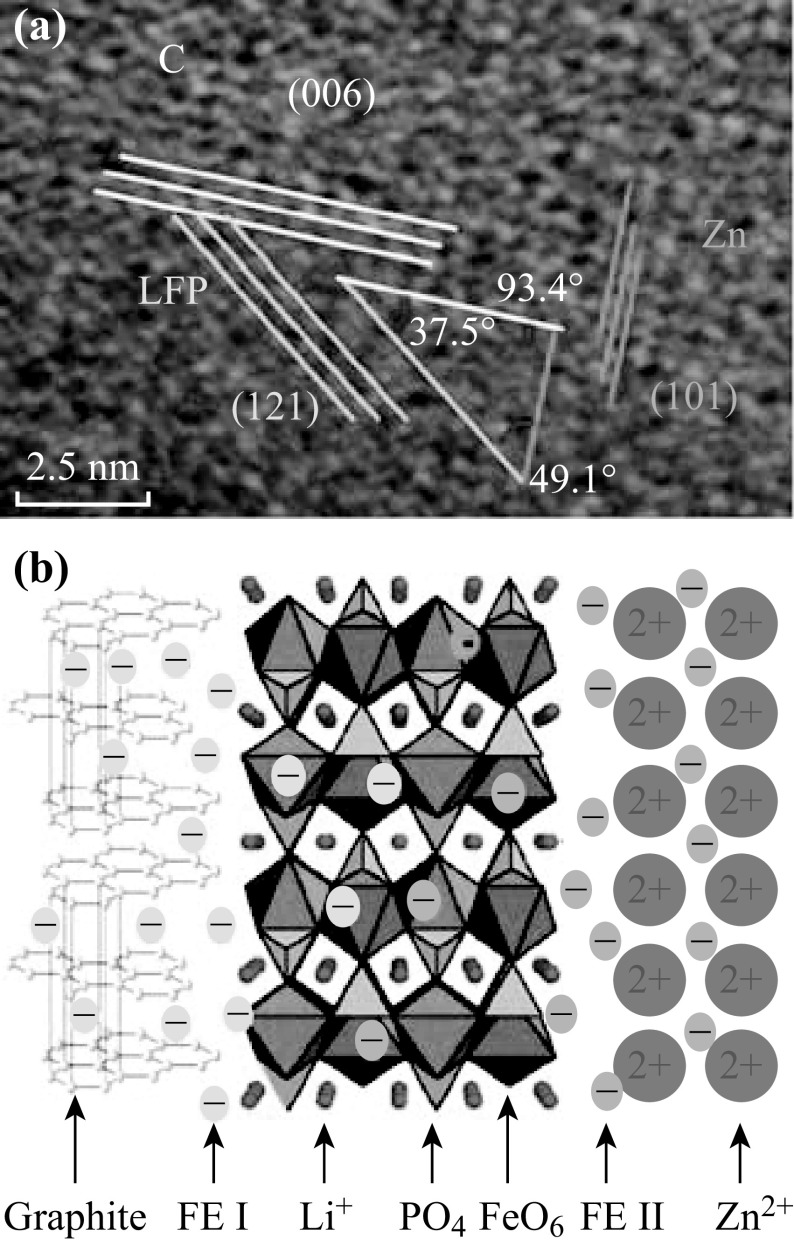
**a** An enlarged TEM image of LFP/C_ZIF-8_. **b** The mechanism of conductivity improvement, FE I represents free electron in graphite, FE II is free electron in metal zinc

Figure 2a exhibits an enlarged TEM image of LFP/C_ZIF-8_, showing clear lattice fringes of LFP (121), Zn (101) and graphite (006). The angle between the (121) plane of LFP and the (101) plane of Zn is 49.1°. The angle is 37.5° between the (121) plane of LFP and the (006) plane of graphite. The angle is 93.4^o^ between (101) plane of Zn and the (006) plane of graphite. It clearly shows the heterostructure of the LFP/C_ZIF-8_ material. This indicates that the nucleation and crystal growth of ZIF-8 nanoparticles occur on the surface of LFP particle. After annealing, the graphite-like carbon and Zn are also formed. Figure 2b illustrates the conductive mechanism of the heterostructure of the LFP/C_ZIF-8_ material. As we know, graphite has good conductivity because it is the ordered *sp* ^2^ carbon and it has free electrons (yellow balls, FE I). Moreover, metal Zn contains a lot of free electrons (green balls, FE II), so it has better conductivity than graphite. As surface coating materials, graphite and Zn are able to improve the conductivity of commercial LFP cathode due to their rich FEs.

**Fig. 3:**
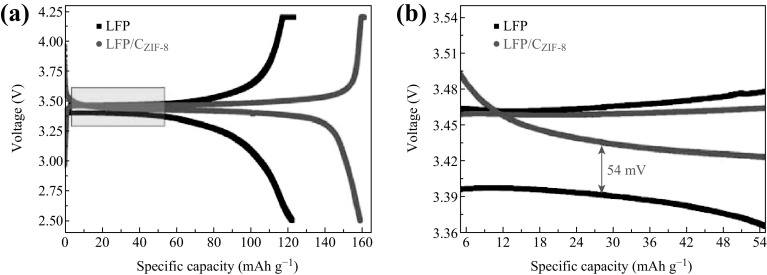
**a** The first charge and discharge curves of cathodes between 2.5 and 4.2 V at 0.1C. **b** The enlarged figure remarked in **a**. The range of voltage is between 3.36 and 3.55 V, the range of specific capacity is between 5.0 and 55.0 mAh g^−1^. The distance between the voltage platforms of two discharge curves is about 54 mV

Figure 3a shows the first charge and discharge curves of LFP and LFP/C_ZIF-8_ cathodes between 2.5 and 4.2 V at 0.1C. The specific capacity is significantly enhanced after surface coating due to the improvement of conductivity. As shown in Fig. 3b, the range of voltage is between 3.36 and 3.55 V, and the range of specific capacity is between 5.0 and 55.0 mAh g^−1^. The distance between the voltage platforms of two discharge curves is about 54 mV. It indicates that the conductivity enhancement leads to a dramatic increase in the discharge voltage platform, which is conducive to the improvement of specific energy.

**Fig. 4:**
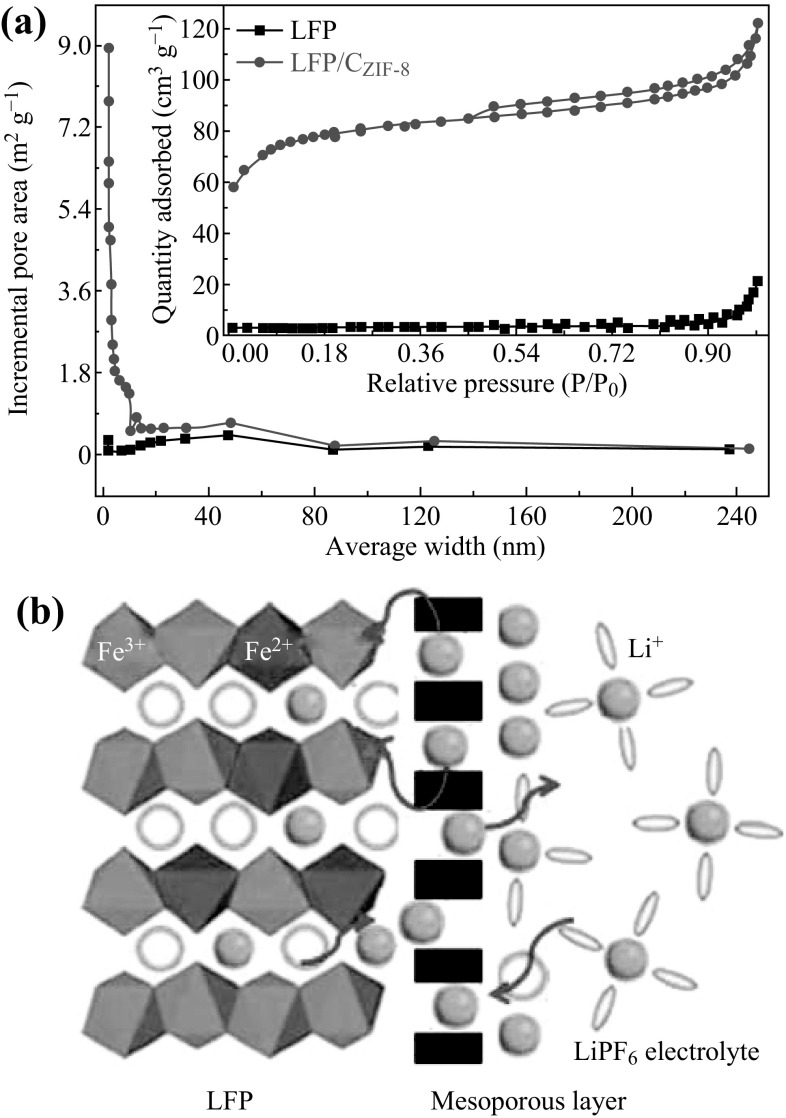
**a** Barrett–Joyner–Halenda pore-size distribution and the isotherm shape of LFP and LFP/C_ZIF-8_. **b** Working mechanism of the coating layer in LFP/C_ZIF-8_

Figure 4a shows the N_2_ adsorption–desorption isotherms of LFP and LFP/C_ZIF-8_. The Barrett–Joyner–Halenda pore-size distribution line chart shows that the LFP/C_ZIF-8_ has a hierarchical pore structure, and the pore-size distribution range centers at 1.9-244.7 nm, while the LFP is almost non-porous. Interestingly, the mesoporous content of LFP/C_ZIF-8_ is the highest in approximate 10 nm, which indicates that there are a large number of 10 nm mesoporous structures in the coating layer. The BET surface areas of LFP and LFP/C_ZIF-8_ are 11.1 and 271.6 m^2^ g^−1^, respectively. The isotherm shapes of the LFP and LFP/C_ZIF-8_ are of the type-III and type-IV patterns [33]. It indicates that LFP and LFP/C_ZIF-8_ possess non-porous and mesoporous characteristics, respectively. Especially, the LFP/C_ZIF-8_ shows the H1 hysteresis loop in the relative pressure (*P*/*P*_0_) range of 0.54–0.90, representing a typical mesoporous feature caused by uniform and simple connecting pores in the surface coating layer [34]. Moreover, the HRTEM image of ZIF-8 annealed at 800 °C is employed to intuitively observe the pore structure (Fig. S3b). The porous size range is similar to the result of N_2_ adsorption–desorption test. It implies that the coating layer of LFP/C_ZIF-8_ possesses rich pore structure. Figure 4b illustrates the working mechanism of the coating layer of the LFP/C_ZIF-8_ cathode materials. Such a mesoporous layer with open interconnected pore structure can significantly increase the permeability of the electrolyte and thus facilitate lithium ion diffusion, which gives a short diffusion path for mass and charge transport. This also leads to a higher degree of freedom for volume change during charge and discharge cycles, and an enhanced lithium ion intercalating behavior.

**Fig. 5:**
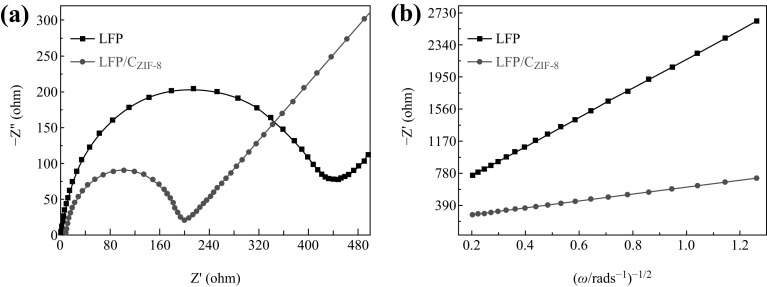
**a** Nyquist plots of LFP and LFP/C_ZIF-8_ cathodes in EIS curves. **b** The plots of impedance as a function of the inverse square root of angular frequency in the Warburg region

The EIS curves of LFP and LFP/C_ZIF-8_ cathodes are shown in Fig. 5a, which are used to compare the ion conductivity after coating. The radius of the semicircle at high-frequency region on the *Z*′-axis is related to the charge transfer resistance (*R*_ct_). The results show that the *R*_ct_ value of LFP/C_ZIF-8_ cathode (188.9 Ω) is obviously less than that of LFP cathode (439.3 Ω). Therefore, the conductivity is improved after coating. Figure 5b shows the slope of inclined line in low frequency representing the Warburg impedance (*W*) of EIS curve, which is used to study the lithium ion diffusion behavior. The slope *σ* can be obtained from the linear fitting of *Z*_real_ versus *ω* ^−1/2^. Using *σ* value, it is possible to calculate the lithium diffusion coefficient of the cathode [35]. The calculation results (Table S1) show that the lithium diffusion coefficient of LFP/C_ZIF-8_ cathode (1.1708 × 10^−13^ cm^2^ s^−1^) is about 20 times that of LFP cathode (6.7553 × 10^−15^ cm^2^ s^−1^). It indicates that the lithium diffusion coefficient is also improved after coating.

Moreover, we investigate the effects of annealing temperature and the ratio of LFP/methanol on the ion conductivity by using EIS test (Fig. S6; Table S1). We find that the increase of the coating material is beneficial to the enhancements of the ion conductivity and lithium diffusion coefficient (Fig. S6a, c). And the LFP/C_ZIF-8_ cathode synthesized at 800 °C delivers the highest conductivity and lithium diffusion coefficient (Fig. S6b, d). The Raman spectra (Fig. S7) show that the peak intensity ratio of the G-band and the D-band (*I*_G_/*I*_D_) is the highest when the annealing temperature is 800 °C. The results suggest that the 800 °C annealed sample has the largest degree of graphitization without damaging the coating layer. Therefore, 800 °C is the optimized annealing temperature with the highest conductivity.

**Fig. 6:**
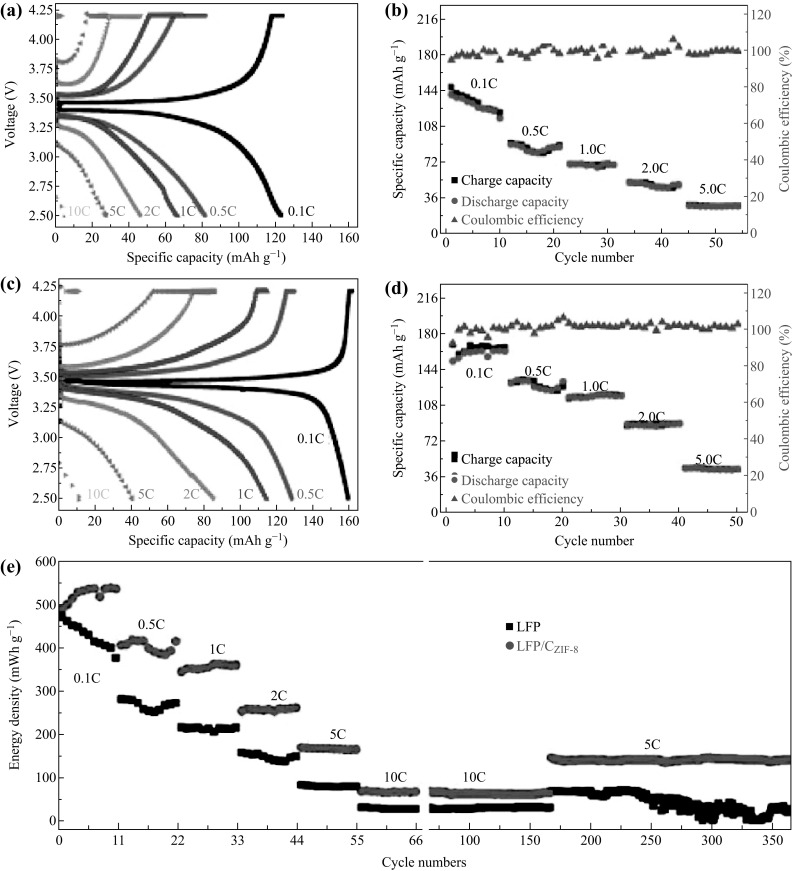
First charge-discharge curves of the LIBs with the **a** LFP and **c** LFP/C_ZIF-8_ at different rates. Capacity retention and Coulombic efficiency of the LIBs with the **b** LFP and **d** LFP/C_ZIF-8_ for 50 cycles at different rates. **e** The discharge specific energy of different cathode samples for 10 cycles at 0.1–10.0C rates, respectively, then for 100 and 200 cycles at 10.0 and 5.0C rates, respectively

The C rate performances and cycle stability of cathode-active materials are shown in Fig. 6. Comparing LFP/C_ZIF-8_ cathode (Fig. 6a) with LFP cathode (Fig. 6c), the discharge specific capacities of LFP/C_ZIF-8_ cathode are all higher than that of LFP cathode at different C rates. The capacity of LFP/C_ZIF-8_ cathode is 159.3 mAh g^−1^ at 0.1C, while that of LFP is 122 mAh g^−1^. Similarly, the capacity retention rates of LFP/C_ZIF-8_ cathode (Fig. 6b) are also higher at all C rates compared with LFP cathode (Fig. 6d). The values of the capacity retention rates are 96.1 and 101.0% before (LFP) and after (LFP/C_ZIF-8_) coating at 2.0C, respectively. The electrochemical performances of LFP active material are significantly improved after coating by C_ZIF-8_.

The discharge specific energies of LFP and LFP/C_ZIF-8_ cathodes’ active materials are shown in Fig. 6e. The discharge specific energies at different C rates are significantly improved, and the average value of LFP/C_ZIF-8_ cathodes’ discharge specific energy is 355.3 mWh g^−1^ at 1.0C, while that of LFP is 212.7 mWh g^−1^. Moreover, the discharge specific energy retention rate of LFP/C_ZIF-8_ cathode is approximate 99% after 200 cycles at 5.0C, while LFP’s discharge specific energy retention rate is only 40%. These results are attributed to the synergy improvements of voltage platform, specific capacity, and freedom degree for volume change after surface coating.

## Conclusions

In summary, we studied the surface modification of commercial LiFePO_4_ (LFP) by utilizing ZIF-8 and synthesized the LFP/C_ZIF-8_ cathodes by growth and carbonization of ZIF-8 on the surface of LFP. The coating layer with metal Zn and graphite-like carbon is about 10 nm. Using as the cathode material, LFP/C_ZIF-8_ clearly improves the conductivity, the lithium ion diffusion coefficient, and the degree of freedom for volume change. Therefore, LFP/C_ZIF-8_ delivers a discharge specific capacity of 159.3 mAh g^−1^ at 0.1C and a discharge specific energy of 141.7 mWh g^−1^ after 200 cycles at 5.0C (the retention rate is approximately 99%).

Although we demonstrated that ZIF-8 was a better surface modification material for commercial LIB cathodes, several key aspects still need to be explored in the future. (1) The theoretical description and prediction of the mechanism of the enhancement of electrochemical performances should be established in future studies. (2) It is necessary to further study the controllability of the ratio of LFP, graphite, and zinc. Potentially, our approach for modifying LFP cathode materials could also be applied in modifying other cathodes for commercial LIBs.

## Electronic supplementary material

Below is the link to the electronic supplementary material.

Supplementary material 1 (PDF 1192 kb)
