# Supplementary material for: The Surface Coating of Commercial LiFePO4 by Utilizing ZIF-8 for High Electrochemical Performance Lithium Ion Battery
Source: Nanomicro Lett. 2017 Sep 25;10(1):1. doi: 10.1007/s40820-017-0154-4 (PMC6199046; doi:10.1007/s40820-017-0154-4)
Supplement: Supplementary file 1 — Supplementary material 1 (PDF 1192 kb) [file 40820_2017_154_MOESM1_ESM.pdf]

Supporting Information for

The Surface Coating of Commercial $LiFePO_4$ by Utilizing ZIF-8 for High Electrochemical Performance Lithium Ion Battery

XiaoLong Xu[1], CongYu Qi[1] (co-first author), ZhenDong Hao[1], Hao Wang[1,*], JinTing Jiu[2], JingBing Liu[1], Hui Yan[1], Katsuaki Suganuma[2]

[1]The College of Materials Science and Engineering, Beijing University of Technology, Beijing 100124, People's Republic of China

[2]The Institute of Scientific and Industrial Research, Osaka University, Osaka, Japan

*Corresponding author. E-mail: haowang@bjut.edu.cn

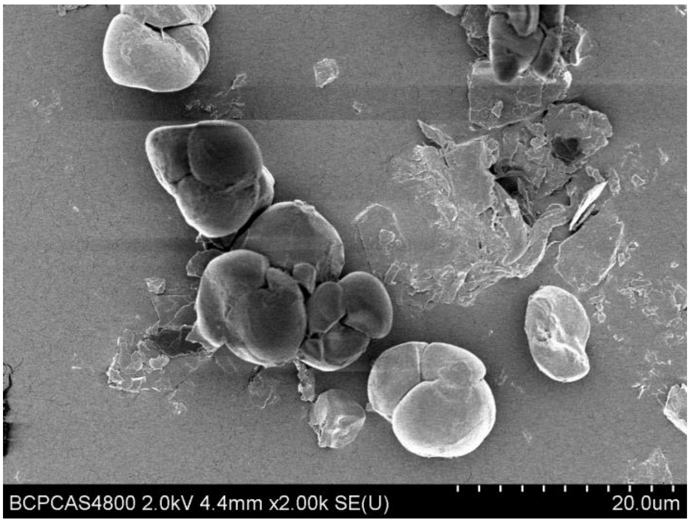

Fig. S1 SEM image of the micron-scale commercial LFP particles

The main crystal compositions of all samples are analyzed by XRD (Fig. S2). The results show that the peaks of all samples are indexed to the olivine ($LiFePO_4$) phase (PDF No. 40-1499), which indicates that the surface coating is not able to alter the crystal structure of LFP.

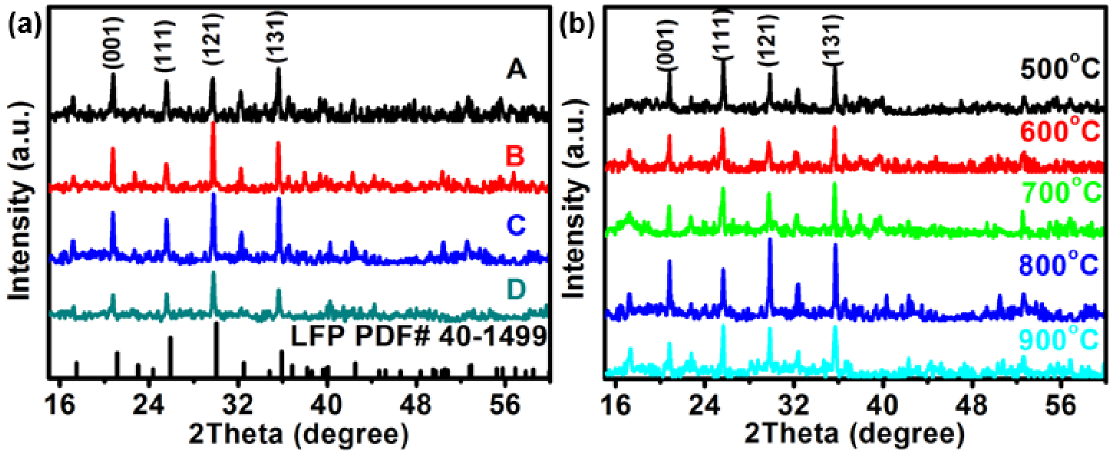

Fig. S2 XRD patterns of **a** the LFP and LFP/C_{ZIF-8} samples synthesized with different ratio of LFP : methanol at 800 °C, and **b** the different LFP/C_{ZIF-8} samples synthesized at different temperature. A, B, C and D represent that the ratios of LFP : methanol are 8 g : x mL (x= 0, 188, 282, 376). The peaks are indexed to the olivine ($LiFePO_4$) phase (PDF No. 40-1499, Pmnb)

Figure S3a shows the XRD curves of ZIF-8, which is synthesized using the same method without LFP. All peaks are consistent with the ZIF-8 nanocrystals. The as-obtained ZIF-8 was annealed at 800 °C under nitrogen flow; all annealing parameters were consistent with the LFP/C_{ZIF-8} sample. Figure S3b displays the HRTEM image of annealed ZIF-8, its porous size range is similar to result of N_2 adsorption–desorption test. The result implies that the coating layer of LFP/C_{ZIF-8} sample possesses rich pore structure.

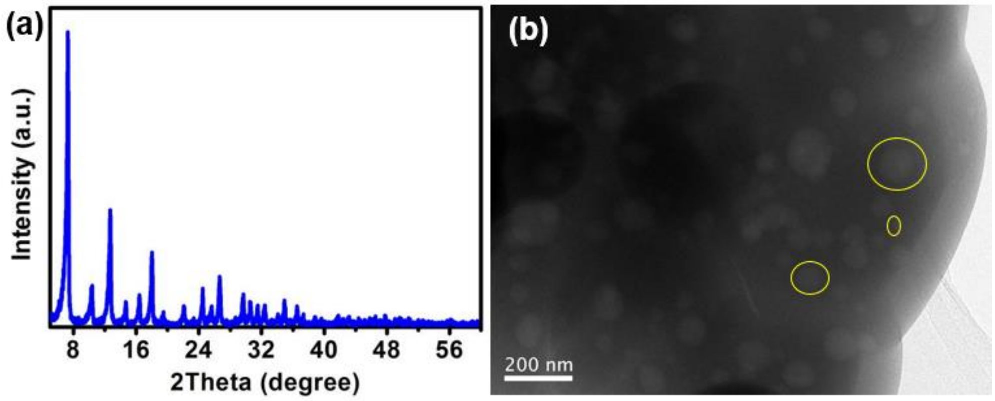

Fig. S3 **a** XRD pattern of ZIF-8. **b** HRTEM image of ZIF-8 annealed at 800 °C

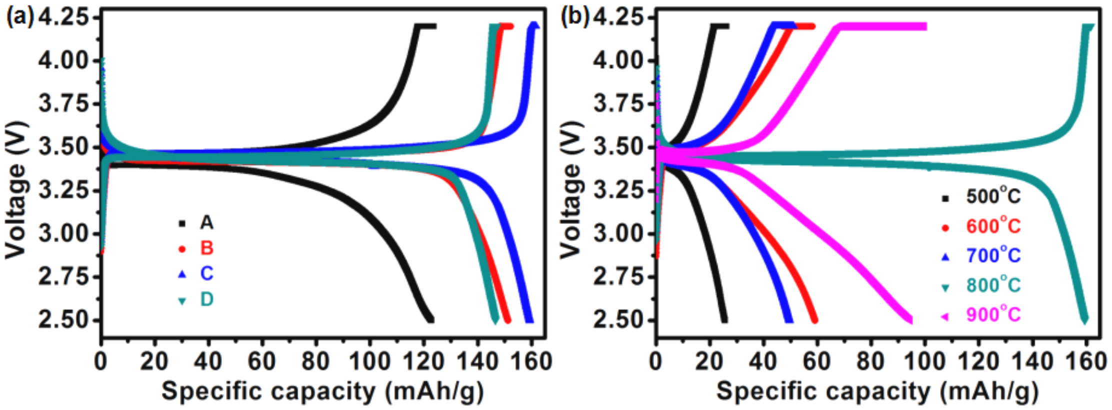

Fig. S4 Electrochemical properties of the different LFP/C_{ZIF-8} cathodes for the coin cells tested between 2.5 and 4.2 V. **a** First charge-discharge curves of the cathodes synthesized with different ratio of LFP : methanol at 800 °C at 0.1 C, the optimal ratio of is 8 g : 282 mL. **b** First charge-discharge curves of the different cathodes synthesized with the optimal ratio at different temperature at 0.1 C, the optimal heat treatment temperature is 800 °C

The electrochemical performances of different LFP/C_{ZIF-8} cathodes synthesized under different conditions are evaluated and compared by charge and discharge tests using CR2032 coin cells (Fig. S4). We found that the LIBs with the LFP/C_{ZIF-8} cathode synthesized with the LFP/methanol ratio of 8 g : 282 mL at 800 °C delivers a specific discharging capacity of 159.3 mAh g^{-1} at 0.1 C, which is much higher than the capacity of LFP without coating (122.8 mAh g^{-1}). All cells exhibit one voltage plateau with discharge voltage near 3.4 V, corresponding to the electrochemically active redox of Fe^{2+}/Fe^{3+}. It also indicates that the surface coating by utilizing ZIF-8 does not alter the crystal structure of LFP.

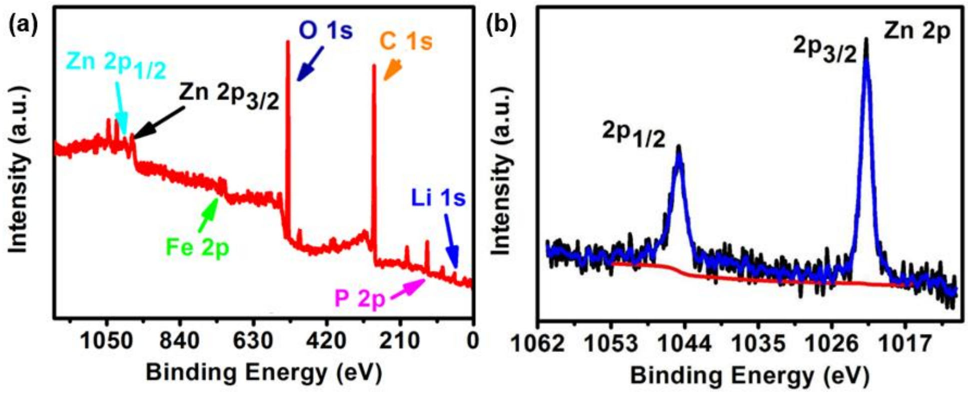

Fig. S5 a XPS survey spectra of LFP/C_{ZIF-8}. **b** The high-resolution XPS spectra of elemental zinc

XPS technique is performed to figure out the surface properties of LFP/C_{ZIF-8} as shown in Fig. S5. The survey spectrum in Fig. S5a indicates the existences of Zn, Fe,

O, C, P, and Li elements in LFP/C_{ZIF-8}. The high-resolution spectra of Zn 2*p* shown in Fig. S5b can be fitted into two peaks at 1021.78 eV (2*p*3/2) and 1044.88 eV (2*p*1/2), a peak splitting of 23.1 eV. It assures the presence of elemental zinc [1], which is beneficial to enhance the conductivity of LFP/C_{ZIF-8}.

We investigate the effects of annealing temperature and the LFP/methanol ratio on the conductivity and lithium diffusion coefficient of LFP/C_{ZIF-8} cathode by employing EIS test (Fig. S6). In Fig. S6a, b, each EIS curve has an intersection with the *Zreal* axis, a semicircle and an oblique line. The intercept in the high frequency region is related to the ohmic series resistance (R_e). It includes the interparticle contact resistance, electrolyte resistance and other physical resistances between the electrolyte and electrode. The radius of the semicircle at high frequency region on the *Z′*-axis is related to the charge transfer resistance (R_{ct}). Figure S6a displays the EIS curves of the cathodes synthesized with different ratio of LFP : methanol at 800 °C. A, B, C, and D represent that the ratios of LFP : methanol are 8 g : x mL (x= 0, 188, 282, 376). The changes of R_{ct} values suggest that the conductivity of LFP/C_{ZIF-8} cathode is improved with the increase of the coating material within a certain range. Figrue S6b shows the different LFP/C_{ZIF-8} samples synthesized at different temperature. The R_{ct} value of the sample synthesized at 800 °C is the smallest, which suggests the optimized annealing temperature of the conductivity is 800 °C.

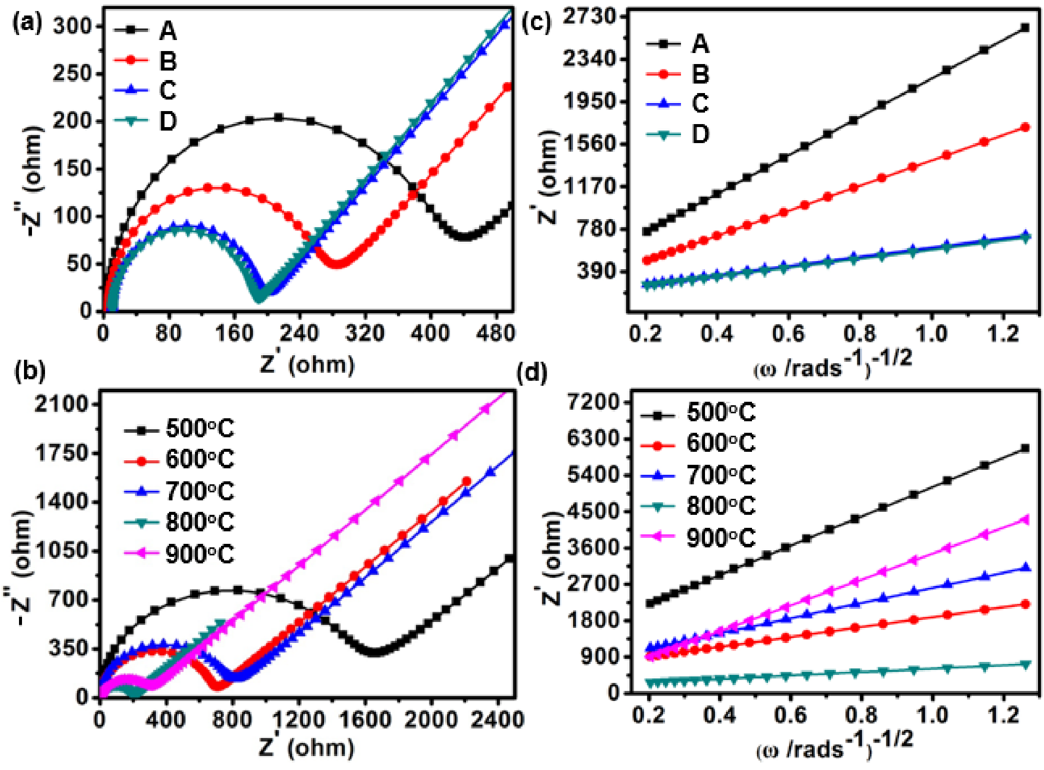

Fig. S6 a, b Nyquist plots of the different cathodes in EIS curves. **c, d** The plots of impedance as a function of the inverse square root of angular frequency in the Warburg region

The slope of inclined line in low frequency represents the Warburg impedance (*W*), which is associated with lithium ion diffusion in LFP/C_{ZIF-8} cathodes (Fig. S6c, d). The diffusion coefficient (*D*) of lithium ions can be calculated from the plots in the low frequency region using Eq. S1,

$$D = \frac{R^2T^2}{2A^2n^4F^4C^2\sigma^2} \qquad \text{(S1)}$$

Where *R* is the gas constant, *T* is the absolute temperature, *A* is the surface area of the cathode, *n* is the number of electrons per molecule during oxidization, *F* is the Faraday constant (96,486 C mol^{-1}), *C* is the concentration of lithium ion, and σ is the Warburg factor which obeys the following relationship:

$$Z_{real} = R_e + R_{ct} + \sigma\omega^{-1/2} \qquad \text{(S2)}$$

Where ω is angle frequency. Figure S6c, d shows the linear fitting of Zreal vs. $\omega^{-1/2}$, from which the slope σ can be obtained. Using this σ value it is possible to calculate the lithium diffusion coefficients of the material. The calculation values indicate that the diffusion coefficient (*D*) is improved with the increase of the coating material. And the optimized annealing temperature is 800 °C.

Table S1 The R_{ct}, σ and *D* values of different samples derived from the EIS in Fig. S6

Sample	R_{ct} (Ω)	σ	*D* ($cm^2\ s^{-1}$)
A	439.3	1766.48	6.7553×10^{-15}
B	284.9	1155.13	1.5798×10^{-14}
C	199.9	424.31	1.1708×10^{-13}
D	188.9	412.13	1.2411×10^{-13}
500	1666.3	3634.46	2.1456×10^{-15}
600	705.5	1228.49	1.3968×10^{-14}
700	818.4	1876.16	5.9886×10^{-14}
800	199.9	424.31	1.1708×10^{-13}
900	229.0	3210.53	2.0451×10^{-15}

The degree of graphitization of the carbon material is closely related to the annealing temperature, thus, the annealing temperature was optimized by Raman test analysis (Fig. S7). The Raman spectra present two strong peaks at about 1350 cm^{-1} (D-band) and 1600 cm^{-1} (G-band). The D-band corresponded to the defects and disordered portions of carbon, while the G-band is associated with the ordered sp^2 carbon [2]. The peak intensity ratio of the G-band and the D-band (I_G/I_D) provided a useful index to estimate the degree of graphitization in carbon materials. The I_D/I_G ratio indicates that the carbonized ZIF-8 in the synthesized sample retain a high

graphitic crystallinity. The results show that the peak intensity ratio of the G-band and the D-band (I_G/I_D) increases with increasing temperature. However, two strong peaks disappear when annealed at 900 °C. It may be attributed to the melting of LFP and the volatilization of carbon [3]. The results indicate that 800 °C annealed samples have the largest degree of graphitization.

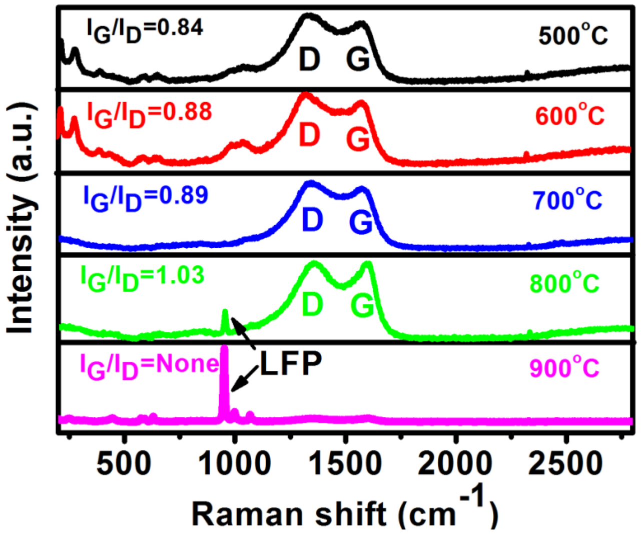

Fig. S7 Raman patterns of the LFP/C_{ZIF-8} samples annealed at different temperatures

References

[1] M. Zubair, A. Razzaq, C.A. Grimesc, S. IlIna, Cu_2ZnSnS_4 (CZTS)-ZnO: A noble metal-free hybrid Z-scheme photocatalyst for enhanced solar-spectrum photocatalytic conversion of CO_2 to CH_4. J. CO_2 Utilization **20**, 301-311 (2017). doi:10.1016/j.jcou.2017.05.021

[2] Z.P. Ma, G.J. Shao, Y.Q. Fan, G.L. Wang, J.J. Song, T.T. Liu, Tunable morphology synthesis of $LiFePO_4$ nanoparticles as cathode materials for lithium ion batteries. ACS Appl. Mater. Interfaces **6**, 9236-9244 (2014). doi:10.1021/am501373h

[3] Z.P. Ma, Y.Q. Fan, G.J. Shao, G.L. Wang, J.J. Song, T.T. Liu, In situ catalytic synthesis of high-graphitized carbon-coated $LiFePO_4$ nanoplates for superior Li-ion battery cathodes. ACS Appl. Mater. Interfaces **7**, 2937-2943 (2015). doi:10.1021/am5084368
